# Understanding Food Waste, Food Insecurity, and the Gap between the Two: A Nationwide Cross-Sectional Study in Saudi Arabia

**DOI:** 10.3390/foods10030681

**Published:** 2021-03-23

**Authors:** Nora A. Althumiri, Mada H. Basyouni, Ali F. Duhaim, Norah AlMousa, Mohammed F. AlJuwaysim, Nasser F. BinDhim

**Affiliations:** 1 Sharik Association for Health Research, Riyadh 13326, Saudi Arabia; mada.basyouni@sharikhealth.net (M.H.B.); 2170001797@iau.edu.sa (N.A.); 216006361@student.kfu.edu.sa (M.F.A.); nasser.bindhim@sharikhealth.net (N.F.B.); 2 Ministry of Health, Riyadh 11176, Saudi Arabia; 3 Saudi Food and Drug Authority, Riyadh 13513, Saudi Arabia; afduhaim@sfda.gov.sa; 4 Public Health Department, Imam Abdulrahman Bin Faisal University, Dammam 31441, Saudi Arabia; 5 Pharmacy College, King Faisal University, AlAhsa 31982, Saudi Arabia; 6 College of Medicine, Alfaisal University, Riyadh 11533, Saudi Arabia

**Keywords:** food security, food waste, household, food insecurity, Saudi Arabia

## Abstract

Background: Food waste and food insecurity may co-exist in various balances in developing and developed countries. This study aimed to explore the levels of food waste and food insecurity, the factors associated with them, and their relationships at the household and individual levels in Saudi Arabia. Methods: This study was a nationwide cross-sectional survey conducted via computer-assisted phone interviews in January 2021. Quota sampling was utilized to generate balanced distributions of participants by gender across all the administrative regions of Saudi Arabia. Data collection included household demographics, food waste and disposal, the Food Insecurity Experience Scale (FIES), and the Household Food Insecurity Access Scale (HFIAS). Results: Out of the 2807 potential participants contacted, 2454 (87.4%) completed the interview. The mean age was 31.4 (SD = 11.7; range = 18–99) and 50.1% were female. The weighted prevalence of uncooked food waste in the last four weeks was 63.6% and the cooked food waste was 74.4%. However, the food insecurity weighted prevalence at the individual level (FIES) was 6.8%. In terms of food insecurity at the household level (HFIAS), 13.3% were in the “severely food insecure” category. Moreover, this study found that “moderately food insecure” households were associated with an increased likelihood to waste uncooked food (relative risk (RR) = 1.25), and the “mildly food insecure” (RR = 1.21) and “moderately food insecure” (RR = 1.17) households were associated with an increased likelihood to waste cooked food. However, “food secure” households were associated with a decreased likelihood to waste cooked food (RR = 0.56). Finally, this study identified four household factors associated with food waste and three household factors that were associated with “severe food insecurity.” Conclusions: This first national coverage study to explore food waste and food insecurity at the individual level and household level, identified household factors associated with food waste and food insecurity and identified new associations between food waste and food insecurity in Saudi Arabia. The associations found between food waste and food insecurity are potential areas of intervention to reduce both food waste and food insecurity at the same time, toward achieving the Sustainable Development Goal (SDG) targets related to food waste and food security.

## 1. Background

As Saudi Arabia imports around 80% of its food needs and this trend is expected to increase, the importance of food waste and food insecurity as concepts are expected to grow exponentially in Saudi Arabia in the near future [1,2,3]. This importance is stimulated by the global coronavirus disease 2019 (COVID-19) pandemic, which has affected the food supply chain and is expected to have a long-term effect on food production and the supply chain globally [4,5,6]. Although Saudi Arabia mainly relies on food imported from all around the globe, due to limited agricultural production, high levels of food waste are still occurring [7,8].

Despite this local context, food waste is a global issue. Food waste reduction is one of the global Sustainable Development Goal (SDG) targets [9]. Target 12.3 of the SDGs aims to halve the global food waste per capita at the retail and consumer levels and to reduce food losses along the production and supply chains, including post-harvest losses, by 2030 [9]. Food waste and food insecurity may coexist in various balances in developing and developed countries [10,11,12]. Surplus food management and the reduction of food waste are increasingly acknowledged as being levers for the mitigation of food insecurity, as both surplus food reduction at the source and its recovery for human consumption are critical elements in the local and global food security effort [11].

In terms of the definitions of food waste and food security, food waste—which occurs at the end of the food chain (retail and final consumption)—is acknowledged to be a huge problem worldwide, even though the definition of various terms and information collection processes are not yet well harmonized [1,11]. However, according to the Food and Agriculture Organization (FAO), food security exists when “all people, at all times, have physical and economic access to sufficient, safe and nutritious food to meet their dietary needs and food preferences for an active and healthy life” [13]. 

Food waste at the consumption (household and individual) level can be categorized into fresh or cooked food waste and unprepared or uncooked food, such as canned or frozen food waste. It also can be categorized based on food type. In Saudi Arabia, a national study conducted in 2018 led by the Saudi Grains Organization to estimate food losses and food waste found that waste was estimated at 2.33 million tons, which represents 18.9% of the volume of the targeted food groups in the study [8]. Most importantly, 57% of the food waste was found to happen at the consumption level [8]. The most wasted types of food in the study were rice at 31%, bread at 25%, meat at 19%, and fruits and vegetables at 16% [8].

Nevertheless, food security rests on three pillars: Food availability (existence of sufficient quantities), food access (households are able to obtain the quantities required), and food utilization (appropriate nutrition and hygiene) [14]. In terms of food insecurity in Saudi Arabia, the FAO report Near East and North Africa, Regional Overview of Food Security and Nutrition used the Food Insecurity Experience Scale (FIES) and stated that severe food insecurity in Saudi Arabia between 2015 and 2017 was 8.1%. However, one study looked at food insecurity during the COVID-19 pandemic in Saudi Arabia using an online convenience sample recruited via social media platforms and used the Household Food Insecurity Access Scale (HFIAS) and found that the overall prevalence of food insecurity was 37.7% in the study sample, with 17.0% of participants experiencing moderate and severe food insecurity [15]. As this is the only study exploring food security on a household level in Saudi Arabia, the prevalence is very high and alarming. 

Although few previous efforts have explored food waste at the consumption level and food insecurity separately, it is important to explore them on a large national level together to bring more insight into the relationship between food waste and food insecurity. Thus, this study aimed to explore the levels of food waste and food insecurity, the factors associated with them, and their relationships at the household and individual levels in Saudi Arabia. The remainder of this paper is structured as follows. The main section presents the method used to conduct this study, the survey question, and the outcomes measured. Section 3 then presents the results based on the objectives of this study. Section 4 provides a summary of the results and a discussion of the important points.

## 2. Method

### 2.1. Study Design

For this study, we conducted a nationwide cross-sectional survey in Saudi Arabia via computer-assisted phone interviews in January 2021.

### 2.2. Sampling and Sample Size

A proportionate quota-sampling procedure was used to obtain an approximately balanced allocation of participants based on gender across the 13 administrative regions of Saudi Arabia (Ar Riyadh, Macca Al Mukaramah, Eastern Province, Asir, Baha, Jazan, Najran, Al Madinah, Al Qassim, Hail, Tabuk, Northern Borders, and Al Jouf), leading to a total of 26 quotas. The sample size was generated using a medium effect size at 0.30, with an 80% sampling power and a 95% confidence interval (CI), to compare regions at the gender level [16]. Therefore, 94 participants were needed in each quota, and a total sample size of 2444 participants was required in this research project [16]. The total sample size in each administrative region was 190 participants at an approximately 0.20 effect size [16]. This study used “QPlatform”, an electronic data collection platform, which has eligibility and sampling functions, to manage the sample distribution process and to prevent human bias in the sampling procedure [17]. Two factors, gender and region, were used to determine the adherence to the sampling quota.

### 2.3. Participant Recruitment

In this study, we recruited Saudi residents, Arabic-speaking adults (≥ 18 years old). We used a random phone number list that was obtained from the Sharik Association for Health Research (Sharik research participants’ database) to identify prospective participants [18]. The Sharik research participants’ database includes individuals who provided consent and are willing to participate in future studies. The database includes more than 75,000 potential research participants distributed across the 13 administrative regions of Saudi Arabia and continues to expand [18]. Potential participants were called up to three times. If there was no response, the number of a new participant with similar demographical characteristics was generated from the Sharik database. After explaining the study to the potential participant, consent to participate in the study was obtained, and the interviewer assessed the eligibility of each participant on the data collection platform, based on the gender and region quota completion criteria. Once the sampling quota was complete, recruitment ceased automatically [17]. For the household-related questions, the participant responded for the entire household.

### 2.4. Survey Design and Outcome Measures

The survey was divided into five sections.

1. The demographics section. This section included age, gender, region, educational level, household’s net income, number of people living in the household, number of children living in the household, elderly people living in the household, and social support status.
2. The household food waste of uncooked items (such as canned food or fresh vegetables). In this section, we asked the participants if they had wasted any uncooked food items within the last four weeks and the frequency of such behavior. If the household had wasted any uncooked items, then they were directed to answer three more questions about the reason for the food waste, the type of food, and how/where the items were wasted.

Food waste for uncooked food items was calculated for the overall sample and categorized based on the reason of food waste, type of food, and how/where the items were wasted.

3. The household food waste of cooked items. In this section, we asked the participants if they had wasted any cooked food items within the last four weeks and the frequency of such behavior. If the household had wasted any cooked food items, then they were directed to answer four more questions about the reason for the food waste, the source of the cooked food, the type of food, and how/where the items were wasted.

Food waste for cooked food items was calculated for the overall sample and categorized based on the reason for the food waste, the source of the cooked food, the type of food, and how/where the items were wasted.

4. The individual food insecurity experience measured via the FIES [19,20]. The FIES was the first tool to be used to measure food insecurity at the individual level globally and was validated by the Food and Agriculture Organization (FAO) in 151 countries, including in Saudi Arabia [21]. The FIES has eight questions, each of which is asked with a recall period of 12 months [19,20]. Respondents answer yes/no to the eight questions and the responses are aggregated to provide raw scores ranging from 0 to 8. Food insecurity is then classified into three categories: (1) Food secure (FS) with raw scores = 0–3; (2) moderate food insecurity (MFI) with raw scores = 4–6; (3) severe food insecurity (SFI) with raw scores = 7–8 [19,20].
5. Household food insecurity was measured via the HFIAS for measurement of food access [22]. The HFIAS is an adaptation of the approach used to estimate the prevalence of food insecurity in the United States annually [22]. The scale was validated in the Arabic language and has been widely used by United Nations countries to measure household food insecurity [22,23,24]. The HFIAS has nine questions, each of which is asked with a recall period of four weeks [22]. If the respondent answers “yes” to an occurrence question, a frequency question is asked to determine whether the condition happened 1 = rarely (once or twice), 2 = sometimes (three to ten times), or 3 = often (more than ten times) in the past four weeks [22]. We used the interviewer instructions and questionnaire administration guide recommended by the Food and Nutrition Technical Assistance III Project [22]. Four indicators were used to determine the characteristics of and changes in household food insecurity (access) in the surveyed population, including (1) household food insecurity access-related conditions, (2) household food insecurity access-related domains, (3) household food insecurity access scale scores, and (4) household food insecurity access prevalence. The calculation method for each indicator is explained in detail in the Food and Nutrition Technical Assistance III Project guide, version 3 [22].

We conducted several rounds of linguistic validation to ensure clarity and understanding of the survey questions via focus groups, where group members were asked to review and discuss survey questions and answers. Based on the outcomes of linguistic validation procedures, the survey was further edited, and the final version was approved.

### 2.5. Data Analysis

Descriptive analysis including (proportions, mean, median, etc.) were used to describe the variables. The prevalence of all variables including food waste, reasons, types, and disposal method by the HFIAS and FIES were weighted to equal the adult population within regions in Saudi Arabia, according to the General Authority of Statistics 2017 Census Report [25]. Bivariate analysis was used to compare the categorical variables. To identify the household factors associated with food waste and severe food insecurity, a logistic regression model, including household characteristic variables including monthly household income, total people living in the household, number of children living in the household, elderly family members living in the household, and receipt of social benefits or aids, was used. 

## 3. Results

Out of the 2807 potential participants contacted, 2454 (87.4%) completed the interview. The mean age was 31.4 (SD = 11.7; range = 18–99) and 50.1% were female. Table 1 shows the participants’ demographics and household characteristics. 

### 3.1. Food Waste

The weighted national prevalence of uncooked food waste in the last four weeks was 63.6%, while for the cooked food waste, it was 74.4%. Table 2 shows food waste, reasons, types, and disposal method in the weighted sample for uncooked and cooked food. 

### 3.2. Individual Food Insecurity Experience

The individual food insecurity experience analysis showed that 2281 (93.2%) were in the FS category, 122 (5.0%) were in the MFI category, and 44 (1.8%) were in the SFI category.

### 3.3. Household Food Insecurity Access

Table 3 shows the results of two HFIAS indicators: (1) Household food insecurity access-related conditions and (2) household food insecurity access-related domains. The weighted average Household Food Insecurity Access Scale score was 1.6 (SD = 3.9; range = 0–27). 

In terms of the household food insecurity access prevalence in the weighted sample, 1815 (74.2%) were in the “food secure” category, 226 (9.2%) were in the “mildly food insecure” category, 104 (4.2%) were in the “moderately food insecure” category, and 325 (13.3%) were in the “severely food insecure” category.

#### 3.3.1. Factors Associated with Food Waste

The regression model showed that only two household factors were associated with uncooked food waste. Receiving social benefits or aids (odds ratio (OR) = 1.35; 95% CI = 1.04–1.75; *p* = 0.025) was significantly associated with increasing the likelihood of uncooked food waste compared to those not receiving social benefits in the model. A household income of less than SAR 5000 (OR = 0.62; 95% CI = 0.46–0.85; *p* = 0.003) and SAR 5001 to 8000 (OR = 0.61; 95% CI = 0.46–0.81; *p* = 0.001) was significantly associated with decreasing the likelihood of uncooked food waste compared to the highest income group in the model.

In terms of cooked food waste, the regression model showed three associated factors. A household income of SAR 11,001 to 13,000 (OR = 1.48; 95% CI = 1.01–2.16; *p* = 0.041) and SAR 13,001 to 16,000 (OR = 1.68; 95% CI = 1.17–2.43; *p* = 0.005) was significantly associated with increasing the likelihood of cooked food waste compared to the highest income group in the model. Elderly family members living in the household (OR = 1.41; 95% CI = 1.12–1.78; *p* = 0.005) was significantly associated with an increased likelihood of cooked food waste compared to no elderly family members living at home in the model. A total of nine people or more living in the household (OR = 1.91; 95% CI = 1.30–2.85; *p* = 0.001) was significantly associated with increasing the likelihood of cooked food waste compared to those living with zero to two people in the model.

#### 3.3.2. Factors Associated with Severe Food Insecurity 

The regression model showed that only three household factors were associated with severe food insecurity. A household income of less than SAR 5000 (OR = 12.49; 95% CI = 6.47–24.11; *p* < 0.001), SAR 5001 to 8000 (OR = 5.67; 95% CI = 2.93–10.95; *p* < 0.001), SAR 8001 to 11,000 (OR = 3.391; 95% CI = 1.70–6.78; *p* = 0.001), SAR 11,001 to 13,000 (OR = 7.37; 95% CI = 3.71–14.65; *p* < 0.001), and SAR 13,001 to 16,000 (OR = 4.10; 95% CI = 2.01–8.34; *p* < 0.001) was significantly associated with increasing the likelihood of severe food insecurity compared to the highest income group in the model. Elderly family members living in the household (OR = 2.43; 95% CI = 1.70–3.17; *p* < 0.001) was significantly associated with increasing the likelihood of severe food insecurity compared to no elderly family members living at home in the model. One to four (OR = 1.49; 95% CI = 1.09–8.34; *p* = 0.012) and five or more children living in the household (OR = 3.97; 95% CI = 2.32–6.80; *p* < 0.001) were significantly associated with increasing the likelihood of severe food insecurity compared to no children living at home in the model.

#### 3.3.3. Associations between Food Waste and Food Insecurity 

In terms of uncooked food waste, the chi-square analysis showed no significant differences between uncooked food waste and individual food insecurity in the FIES. In addition, there were no significant differences between uncooked food waste and household food secure, mildly food insecure, and severely food insecure categories of the HFIAS. However, there were significant differences between uncooked food and the household moderately food insecure category of the HFIAS X^2^ (1, *n* = 2447) = 10.5, *p* = 0.001, in which the moderately food insecure participants were associated with increased likelihood of wasting uncooked food (relative risk (RR) = 1.25; 95% CI = 1.25–1.39). 

In terms of cooked food waste, chi-square analysis showed no significant differences between cooked food waste and individual food insecurity in the FIES. In addition, there were no significant differences between cooked food waste and the household severely food insecure category of the HFIAS. However, there were significant differences between cooked food and the household mildly food insecure category of the HFIAS X^2^ (1, *n* = 2447) = 25.9, *p* < 0.001, in which mildly food insecure households were associated with an increased likelihood of wasting cooked food (RR = 1.21; 95% CI = 1.15–1.28). In addition, there were significant differences between cooked food and the household moderately food insecure category of the HFIAS X^2^ (1, *n* = 2447) = 8.3, *p* = 0.004, in which moderately food insecure households were associated with an increased likelihood of wasting cooked food (RR = 1.17; 95% CI = 1.08–1.27). Finally, there were significant differences between cooked food and the household food secure category of the HFIAS X^2^ (1, *n* = 2447) = 8.3, *p* = 0.004, in which food secure households were associated with decreased likelihood of wasting cooked food (RR = 0.56; 95% CI = 0.44–0.70).

## 4. Discussion

This study aimed to explore the levels of food waste and food insecurity, the factors associated with them, and their relationships at the household and individual levels in a nationwide sample of adults from Saudi Arabia. 

The results showed that the weighted prevalence of uncooked food waste in the previous four weeks was 63.6%, and the cooked food waste prevalence was 74.4%. However, the food insecurity weighted prevalence at the individual level was 6.8%. In terms of food insecurity at the household level, 13.3% of participants were in the severely food insecure category. Moreover, this study identified four household factors associated with food waste: Receiving social benefits or aids, household income, elderly family members in the household, and total number of people in the household. In addition, the household fac-tors that were associated with severe food insecurity were household income, elderly family members in the household, and the number of children in the household. Finally, this study explored the association between food insecurity and food waste and found that moderately food insecure households had an increased likelihood of wasting uncooked food. Moreover, the mildly and moderately food insecure households had an increased likelihood of wasting cooked food; however, food secure households had a decreased likelihood of wasting cooked food. 

Our study showed a high level of food waste at the household level in the previous four weeks, with prevalence levels of 63.6% and 74.4%, respectively. These values are higher than the prevalence of general food waste at the household level in the previous four weeks identified in a national survey in 2018 that found a weighted prevalence at 60% [26]. This confirms the previous finding that 57% of food waste happens at the consumption level [8]. Nevertheless, food waste at the consumption level is a global challenge, accounting for 61% of all food loss and food waste in North America and Oceania and 52% in Europe. Moreover, a recent study in South Korea found that approximately 63% of households discharge food waste of less than 500 g a day [27]. Thus, a global effort to combat food waste is urgently needed now more than ever before to protect global food security and food sustainability. 

In addition to the high prevalence of food waste found in this study, the methods of food waste disposal are also a challenge, as the two major methods of disposal found in this study were trash bins and feeding stray animals. As well as being unstainable methods of food waste management, they are also harmful to the environment. However, although there is growing interest in the relegalization of using food waste as animal feed, the practice of feeding stray animals could pose environmental and health threats, especially in residential areas [28,29]. One study identified some major challenges for food waste management in Saudi Arabia, including solid waste segregation, inadequate legislations, well-accepted traditional landfill disposal practices, public attitudes, a lack of awareness, and uncertainty of the acceptability of food waste byproducts [28]. These challenges need to be prioritized and addressed at the national level in the near future. 

Moving on to the prevalence of food insecurity, the prevalence of food insecurity at the individual level in the previous 12 months was slightly lower than the figure generated by the FAO in 2018, which was 8.1% in Saudi Arabia, and was also lower than the average prevalence of food insecurity at the individual level in the Gulf cooperation region, which was found to be 7.6% [29]. However, in terms of the prevalence of food insecurity at the household level in the previous four weeks, the values found in this study were relatively high; however, unfortunately, no previous national-level studies have used the same measurement tool in Saudi Arabia or within the Gulf cooperation region to allow a meaningful comparison. Despite this, it is important to mention that the 4 weeks measured in this study fell in the period of COVID-19 restrictions, including limiting gatherings to 20 people and closing all dine-in restaurants and food facilities in all regions of Saudi Arabia, which may have contributed to the food insecurity recall by the participants. In addition, cooked food banks rely on the use of food left over from restaurants and large events, and thus they could not operate efficiently during this period of restrictions. 

In terms of the factors associated with food waste and food insecurity at the house-hold level, these factors seem logical in a local culture context. One study highlighted that “Food waste patterns are shaped by cultural approaches to the special events and every-day shopping, cooking, and eating. Saudis place high value on generous hospitality; providing ample food is a gesture of welcome” [3]. However, no previous studies con-ducted in Saudi Arabia have explored the associations of household characteristics with food waste or food insecurity to allow a meaningful comparison to be conducted.

However, the associations between food waste and food insecurity are interesting. Although there was no association between food waste and food insecurity at the individual level, there were significant associations between food waste and food insecurity at the household level. One simple explanation might be related to the fact that food waste was measured on a household level, not an individual level. However, the findings showed that food waste is likely to be greater among mildly and moderately food insecure households, while it is lower among food secure households. These findings show that food waste and food insecurity may co-exist in the same household, which represents both a challenge in understanding why this phenomenon is happening and an opportunity to address the food waste issue and to improve food security levels. 

Nevertheless, this study has some limitations. It could be criticized for using quota sampling, which has an associated risk of selection bias, rather than using a random probability sampling approach. However, the costs of probabilistic sampling are significantly greater, and for this project intention and the type of variables, the risk of some (generally low-level) [17,18] bias was considered acceptable [30,31]. In addition, using a proportional large sample with large number of quotas plays an important role in reducing the selection bias [30,31]. Currently, in Saudi Arabia, the only way to generate a random national-level sample is via a household survey, which also has some significant limitations due to sociocultural factors and was not possible during periods of COVID-19 restrictions. However, the recruitment and sampling methods used in this research project have been used successfully in many national projects in Saudi Arabia [32,33,34]. Another point that may represent a limitation in relation to the HFIAS use in this study is that some of the participants may not be the head of the household, however, according to the national authority of statistics in Saudi Arabia, the method nationally adopted of direct contact with the household specifies that it can be through direct contact with the head of the household or any adult member of the household who is familiar with the household affairs [35]. In addition, the HFIAS administration guide followed in this study did not specify which member of the household must be interviewed [22]. 

## 5. Conclusions

This first national coverage study to explore food waste and food insecurity at the household level identified household factors associated with food waste and food insecurity and identified new associations between food waste and food insecurity in Saudi Arabia. The associations found between food waste and food insecurity are potential areas of intervention for simultaneously reducing food waste and food insecurity, which could aid in achieving the SDG targets related to food waste and food security. Some of the interventions that may help include public awareness campaigns of food waste, food security, and food waste management, providing food management educational programs to households receiving social benefits, encouraging investment in the food recycling industry, and reexamining regulations related to the utilization of food waste as animal feed. 

## Figures and Tables

**Table 1:** Participants’ demographics and household characteristics in the sample.

Variable	*n* (%)
Sex	
Female	1230 (50.1)
Male	1224 (49.9)
Education level	
Less than bachelor’s degree	1077 (43.9)
Bachelor’s degree and above	1377 (56.1)
Regions	
Asir	189 (7.7)
Baha	190 (7.7)
Eastern region	189 (7.7)
Hail	191 (7.8)
Jazan	185 (7.5)
Al Jouf	183 (7.5)
Madinah	192 (7.8)
Makkah	191 (7.8)
Najran	190 (7.7)
Northern border	182 (7.4)
Qassim	191 (7.8)
Riyadh	191 (7.8)
Tabuk	190 (7.7)
Monthly household income *	
Less than SAR 5000	385 (15.7)
SAR 5001 to 8000	489 (19.9)
SAR 8001 to 11,000	394 (16.1)
SAR 11,001 to 13,000	290 (11.8)
SAR 13,001 to 16,000	304 (12.4)
SAR 16,001 to 20,000	250 (10.2)
More than SAR 20,000	342 (13.9)
Total people living in the household	
0 to 2	277 (11.3)
3 to 5	779 (31.7)
6 to 8	779 (37.5)
9 and above	478 (19.5)
Number of children living in the household	
0	702 (28.6)
1 to 4	1619 (66.0)
5 and above	133 (5.4)
Elderly family members living in the household	
Yes	637 (26.0)
No	1817 (74.0)
Receiving social benefits or aids	
Yes	364 (14.8)
No	2090 (85.2)

* SAR = USD 3.75.

**Table 2:** Food waste, reasons, types, and disposal method in the weighted sample.

Variable	*n* (%)
Wasted any uncooked food in the last 4 weeks	
Never	892 (36.4)
1 day a week maximum	1041 (42.5)
2 to 3 days a week	411 (16.8)
4 days or more per week	103 (4.2)
Reasons for wasting uncooked food in the last 4 weeks *	
Expired food	1081 (44.2)
Old but not expired food	248 (10.1)
Recalled by authority	74 (3.0)
Caused extreme allergic reaction	87 (3.6)
Caused mild allergic reaction	66 (2.7)
Caused digestive issues	119 (4.9)
We don’t need it anymore	388 (15.9)
Not used for a long time (clearing storage)	663 (27.1)
Other	128 (5.2)
Types of wasted uncooked food in the last 4 weeks *	
Fresh dairy products	864 (35.3)
Long-life dairy products	206 (8.4)
Fruits and vegetables	637 (26.0)
Egg	107 (4.4)
Baby food	94 (3.8)
Meat	93 (3.8)
Canned food	265 (10.8)
Poultry	109 (4.4)
Nuts	66 (2.7)
Fish	51 (2.1)
Long-life juice	129 (5.3)
Soft drinks	114 (4.7)
Eastern sweets	182 (7.5)
Chocolates	131 (5.3)
Uncooked rice	63 (2.6)
Other	227 (9.3)
Disposal methods of uncooked food in the last 4 weeks *	
Trash bin	878 (35.9)
Feeding stray animals	990 (40.5)
Feeding pets	279 (11.4)
Used as compost	57 (2.3)
Food donation to individuals	339 (13.9)
Food recycling	41 (1.7)
Food donation to nonprofit organizations	109 (4.5)
Other	67 (2.7)
Wasted any cooked food in the last 4 weeks	
Never	626 (25.6)
1 day a week maximum	984 (40.2)
2 to 3 days a week	617 (25.2)
4 days or more per week	220 (9.0)
Reasons for wasting cooked food in the last 4 weeks *	
It was more than what we could eat	1168 (47.8)
We do not store cooked food or leftovers	474 (19.4)
We don’t have enough space to store it	135 (5.5)
Spoiled	963 (39.3)
Burned or was not cooked properly	262 (10.7)
Other	145 (5.9)
Types of wasted cooked food in the last 4 weeks *	
Bread and bakeries	916 (37.4)
Pies and pastries	679 (27.7)
Cooked vegetables and seasoned salads	657 (26.8)
Cooked rice	1293 (52.8)
Meat	492 (20.1)
Poultry	763 (31.2)
Fish	184 (7.5)
Egg	167 (6.8)
Grains and legumes	350 (14.3)
Other	196 (8.0)
Disposal methods of cooked food in the last 4 weeks *	
Trash bin	732 (29.9)
Feeding stray animals	1183 (48.3)
Feeding pets	371 (15.2)
Used as compost	64 (2.6)
Food donation to individuals	502 (20.5)
Food recycling	60 (2.5)
Food donation to nonprofit organizations	137 (5.6)
Other	80 (3.3)
Sources of wasted cooked food in the last 4 weeks *	
Restaurant	1166 (47.7)
Home	1633 (66.8)
Local family food business **	257 (10.5)
Neighbors or relatives	205 (8.4)

* Participant can provide more than one answer; ** cooked in the home of a family business entity and delivered by order.

**Table 3:** The weighted prevalence and frequency of each item in the Household Food Insecurity Access Scale (HFIAS).

HFIAS Domains	HFIAS Item	No	Yes (Total)	Frequency of Experience *n* (%)		
		*n* (%)	*n* (%)	Yes (Rarely)	Yes (Sometimes)	Yes (Often)
Anxiety and uncertainty	1. In the past four weeks, did you worry that your household would not have enough food?	2048 (83.7)	399 (16.3)	289 (11.8)	67 (2.7)	43 (1.7)
Insufficient quality	2. In the past four weeks, were you or any household member not able to eat the kinds of foods you preferred because of a lack of resources?	2027 (82.8)	419 (17.2)	253 (10.3)	122 (5.0)	45 (1.8)
	3. In the past four weeks, did you or any household member have to eat a limited variety of foods due to a lack of resources?	2039 (83.4)	408 (16.6)	279 (11.4)	81 (3.3)	48 (2.0)
	4. In the past four weeks, did you or any household member have to eat some foods that you really did not want to eat because of a lack of resources to obtain other types of food?	2144 (87.6)	302 (12.4)	192 (7.8)	83 (3.4)	27 (1.1)
Insufficient food intake	5. In the past four weeks, did you or any household member have to eat a smaller meal than you felt you needed because there was not enough food?	2209 (90.3)	237 (9.7)	148 (6.1)	57 (2.3)	31 (1.3)
	6. In the past four weeks, did you or any household member have to eat fewer meals in a day because there was not enough food?	2186 (89.4)	260 (10.6)	169 (6.9)	63 (2.6)	28 (1.1)
	7. In the past four weeks, was there ever no food to eat of any kind in your household because of lack of resources to get food?	2207 (90.2)	239 (9.8)	148 (6.1)	56 (2.3)	35 (1.4)
	8. In the past four weeks, did you or any household member go to sleep at night hungry because there was not enough food?	2189 (89.5)	257 (10.5)	179 (7.3)	49 (2.0)	29 (1.2)
	9. In the past four weeks, did you or any household member go a whole day and night without eating anything because there was not enough food?	2265 (92.6)	181 (7.4)	123 (5.0)	34 (1.4)	24 (1.0)

## Data Availability

The dataset can be obtained from the Sharik Association for Health Research upon request.
